# Outcomes of dysvascular partial foot amputation and how these compare to transtibial amputation: a systematic review for the development of shared decision-making resources

**DOI:** 10.1186/s13643-017-0433-7

**Published:** 2017-03-14

**Authors:** Michael P. Dillon, Matthew Quigley, Stefania Fatone

**Affiliations:** 1 Discipline of Prosthetics and Orthotics, College of Science, Health and Engineering, La Trobe University, Bundoora, Victoria 3086 Australia; 2 Northwestern University Prosthetics-Orthotics Centre, Feinberg School of Medicine, Northwestern University, 680 N Lake Shore Drive, Suite 1100, Chicago, IL 60611 USA

**Keywords:** Amputation, Partial foot, Mobility, Participation, Function, Quality of life, Mortality, Reamputation, Psychosocial, Pain

## Abstract

**Background:**

Dysvascular partial foot amputation (PFA) is a common sequel to advanced peripheral vascular disease. Helping inform difficult discussions between patients and practitioners about the level of PFA, or the decision to have a transtibial amputation (TTA) as an alternative, requires an understanding of the current research evidence on a wide range of topics including wound healing, reamputation, quality of life, mobility, functional ability, participation, pain and psychosocial outcomes, and mortality. The aim of this review was to describe a comprehensive range of outcomes of dysvascular PFA and compare these between levels of PFA and TTA.

**Methods:**

The review protocol was registered in PROSPERO (CRD42015029186). A systematic search of the literature was conducted using MEDLINE, EMBASE, psychINFO, AMED, CINAHL, ProQuest Nursing and Allied Health, and Web of Science. These databases were searched using MeSH terms and keywords relating to different amputation levels and outcomes of interest. Peer reviewed studies of original research—irrespective of the study design—were included if published in English between 1 January 2000, and 31 December 2015, and included discrete cohort(s) with dysvascular PFA or PFA and TTA. Outcomes of interest were rate of wound healing and complications, rate of ipsilateral reamputation, quality of life, functional ability, mobility, pain (i.e., residual limb or phantom pain), psychosocial outcomes (i.e., depression, anxiety, body image and self-esteem), participation, and mortality rate. Included studies were independently appraised by two reviewers. The McMaster Critical Review Forms were used to assess methodological quality and identify sources of bias. Data were extracted based on the Cochrane Consumers and Communication Review Group’s data extraction template by a primary reviewer and checked for accuracy and clarity by a second reviewer. Findings are reported as narrative summaries given the heterogeneity of the literature, except for mortality and ipsilateral reamputation where data allowed for proportional meta-analyses.

**Results:**

Twenty-nine unique articles were included in the review, acknowledging that some studies reported multiple outcomes. Eighteen studies reported all-cause proportionate mortality. A smaller number of studies reported outcomes related to functional ability (two), mobility (four), quality of life (three), ipsilateral reamputation (six) as well as wound healing and complications (four). No studies related to pain, participation or psychosocial outcomes met the inclusion criteria. Subjects were typically older and male and had diabetes among other comorbidities. More detailed information about the cohorts such as race or sociodemographic factors were reported in an ad hoc manner. Common sources of bias included contamination, co-intervention, or lack of operational definition for some outcomes (e.g., wound healing) as illustrative examples.

**Conclusions:**

Aside from mortality, there was limited evidence regarding outcomes of dysvascular PFA, particularly how outcomes differ between levels of PFA and TTA. Acknowledging that there is considerable uncertainty given the small body of literature on many topics where the risk of bias is high, the available evidence suggests that a large proportion of people with PFA experience delayed wound healing and ipsilateral reamputation. People with TTA have increased risk of mortality compared to those with PFA, which may reflect that those considered suitable candidates for TTA have more advanced systemic disease that also increases the risk of dying. Mobility and quality of life may be similar in people with PFA and TTA.

**Systematic review registration:**

CRD42015029186

**Electronic supplementary material:**

The online version of this article (doi:10.1186/s13643-017-0433-7) contains supplementary material, which is available to authorized users.

## Background

Dysvascular lower limb amputation is a common sequel to advanced peripheral vascular disease resulting in a wide range of adverse health outcomes (e.g., impaired mobility, depression) that often lead to significant disability and reduced quality of life (QoL) [1, 2]. Hence, it is not surprising that the decision to proceed with amputation, even so-called *minor* or partial foot amputation (PFA), is a difficult one.

Helping people make well-informed decisions about PFA has become increasingly important given the shift in types of lower limb amputation performed [3–7]. The incidence of transtibial amputation (TTA) has declined steadily since about the year 2000 [3, 8–11], and there is some evidence that PFA has increased proportionately [3, 6, 10].

The shift to more distal amputation may be seen as a significant improvement given the assumption that more distal amputation results in better mobility [12], improved QoL [13–15], and lower mortality [16–18]. However, PFA has been associated with low rates of healing and complications [13, 14, 19–22] that often lead to ipsilateral reamputation [20, 23–28]. The low rates of healing and high rates of reamputation in people with PFA are disproportionate given that most TTAs heal and only a small proportion require ipsilateral reamputation [24, 28–30].

Some authors [31, 32] have challenged the belief that the high rates of complications and reamputation associated with PFA are worth the benefits, particularly given that key outcomes such as mobility [33, 34] and QoL [35–38] appear to be comparable in people with PFA and TTA. However, closer scrutiny of the evidence is necessary [39–41] given that much of the literature has focused on people with amputations through the midfoot (e.g., transmetatarsal amputation, TMA) and outcomes may be better for people with toe amputations [39, 40]. Similarly, very high rates of wound healing have been reported in some studies of people with PFA—comparable to those for people with TTA—but it is unclear what most influenced the outcomes [19, 42] or whether these outcomes are typical of the broader body of literature.

While research seems to have focused on outcomes related to surgery, mortality, QoL, and mobility, it is also important that decisions about PFA are informed by an understanding of the outcomes related to participation, functional ability, pain, and psychosocial considerations including depression, anxiety, body image, and self-esteem.

A systematic review describing a comprehensive range of outcomes following PFA would help provide the evidence needed for people to make well-informed decisions about the level of PFA and how this compares to TTA, particularly if the results could be used to create shared decision-making resources designed to facilitate and inform discussions between clinicians and patients [43]. Hence, the aims of this systematic review were to:

1. Describe the outcomes of dysvascular PFA with reference to wound healing and complications, ipsilateral reamputation, QoL, functional ability, mobility, pain, psychosocial outcomes, participation, and rate of mortality.
2. Compare these the same outcomes between levels of PFA and TTA.

## Methods

A detailed protocol for this systematic review was registered in PROSPERO (CRD42015029186) and published prior to conduct of the review [44]; hence, a summary of the method has been reported here. The review was reported in accordance with the Preferred Reporting Items for Systematic Reviews and Meta-Analyses (PRISMA) guidelines [45] including a copy of the PRISMA checklist (Additional file 1).

### Search strategy

A systematic search of the literature was conducted using MEDLINE, EMBASE, psychINFO, AMED, CINAHL, ProQuest Nursing and Allied Health, and Web of Science. A list of search terms related to the population (e.g., PFA) and outcomes of interest (e.g., QoL), as well as their synonyms and acronyms, were used in conjunction with wildcards and Boolean operators as part of a title, abstract, and keyword search [44]. Each search strategy was rigorously developed, tested, and refined by comparing the precision and comprehensiveness of the yield to a bank of known articles on each topic [44].

All searches were restricted to the English language given that such restriction does not seem to alter the outcome of systematic reviews and meta-analyses [46, 47]. Searches were also restricted to publications since 1 January 2000, given that changes in treatment practices (e.g., common place use of revascularization prior to, or in conjunction with, amputation) have affected the outcomes since this time as evidenced by changes in the relative risk of amputation [8, 9, 11].

In keeping with the PRISMA guidelines [45], an illustrative search has been presented for one database (Table 1).

**Table 1:** Example search for the CINAHL database to identify quality of-life literature for people with dysvascular partial foot and transtibial amputation

Search	Field code	Search term(s)
1.	MH	“Amputation”
2.	MH	“Amputees”
3.	TI,AB,SU	(amput* AND (major OR lowerlimb* OR “lower limb”* OR “lower extremit*” OR “limb loss” OR LEA OR LLA))
4.	TI,AB,SU	(amput* AND (transtibial OR “trans tibial” OR belowknee OR “below knee” OR (below W2 knee) OR TTA OR BKA))
5.	TI,AB,SU	(amput* AND (minor OR “partial foot” OR Chopart* OR Lisfranc* OR tarsometatarsal OR transmetatarsal OR midtarsal OR “mid tarsal” OR midfoot OR “midfoot” OR ray OR phalangeal OR metatarsophalangeal OR toe* OR transtarsal OR “trans tarsal” OR TMT OR TMA OR MTP OR PFA))
6.		1 OR 2 OR 3 OR 4 OR 5
7.	TI,AB,SU	SF 36 OR SF36 OR “Medical Outcome* Study Short Form*” OR “Medical Outcome* Study Short-Form*” OR “MOS SF 36” OR “MOS SF36” OR “Sickness Impact Profile*” OR “SIP” ((“Trinity Amputation and Prosthe* Experience”) W1 (Survey OR Scale*)) OR TAPES OR “Prosthe* Evaluation Questionnaire” OR PEQ OR “WHO QOL BREF” OR “WHO QOLBREF” OR “WHOQOLBREF” OR ((WHO OR “World Health Organi#ation”) W1 (“Quality of Life BREF” OR “Quality of Life Scale”)) OR “RAND36” OR “RAND 36” OR “Orthotic*and Prosthetic* User* Survey” OR OPUS OR ((“Health Related”) W1 “Quality of Life”) OR HRQOL OR “Life Satisfaction Questionnaire* 9” OR “LiSat 9” OR “Satisfaction With Life Scale” OR SWLS OR “Quality of Well Being” OR QWB* OR “Quality of Life Index” OR QLI OR “EuroQOL*” OR “Euro QOL” OR EQ5D OR “EQ 5D” OR “Assessment of Quality of Life” OR AQoL OR (Orthotic* W2 “prosthetic* user* survey”) OR “Attitude to Artificial Limb* Questionnaire” OR AALQ
8		6 AND 7
9.		Limit 8 to English language
10.		Limit 9 to publication date: 01.01.2000 to 31.12.2015
11.		Limit 10 to peer reviewed, academic journals

Field codes: *MH* Exact major and minor subject headings (MeSH, National Library of Medicine Medical Subject Headings), *TI* Title, *AB* Abstract, *SU* Subject. Asterisk denotes wildcards used to capture truncation or variation of the search terms

For articles that met the inclusion criteria, reference lists were hand searched to ensure that relevant publications were not overlooked. A forward-citation search was conducted using Google Scholar to identify literature published since 1 January 2014, (e.g., early on-line versions of articles) that had not yet been indexed in traditional databases [48–50].

### Data management

Results from each database search were exported into a shared EndNote X7.2.1 library (Thomson Reuters Inc., Philadelphia, PA, USA.) and duplicate records deleted [44]. Full-text copies of articles were retrieved and linked to the relevant EndNote record. Bibliographic information for each reference were exported into an Excel 2013 (Microsoft Corporation Inc., North Ryde, Sydney, Australia) spreadsheet and columns added to record details about exclusion and full-text retrievals. The same spreadsheet was expanded for data extraction and to record details of the critical appraisal [44].

### Selection process

The following criteria were used to determine inclusion:

1. Peer reviewed studies of original research, irrespective of the study design
2. English language
3. Published between 1 January 2000 and 31 December 2015
4. Discrete cohort(s) with dysvascular PFA, irrespective of the level of PFA (aim 1) or PFA and TTA (aim 2)
5. Measured an outcome of interest—rate of wound healing and complications (e.g., dehiscence), rate of ipsilateral reamputation, QoL, functional ability, mobility, pain (i.e., residual limb or phantom pain), psychosocial outcomes (i.e., depression, anxiety, body image, and self-esteem), participation, and mortality rate.

Definitions of TTA and PFA were consistent with the International Standards Organization (ISO) definitions [51], and as such all levels of PFA, including toe amputation, were included, but ankle disarticulation (i.e., Syme’s amputation) was excluded. Articles were included irrespective of the way the outcome of interest was operationally defined or the time point of measurement [44].

Search results were screened by one investigator based on review of the title, abstract, or full-text article as necessary. Following screening, full-text articles were retrieved and independently reviewed by two investigators to confirm inclusion. An additional opinion was sought from a third investigator in cases of disagreement, and discussion continued until consensus.

### Quality appraisal/risk of bias in individual studies

The McMaster Critical Review Forms [52] were used to assess methodological quality and identify sources of bias. The appraisal tool was appropriate for use with a wide variety of study designs [53] and included structured guidelines to reduce the likelihood of errors with use [54]. Results from the quality appraisal were reported in tabular format using Excel and included detailed comments to support the checklist items [44].

### Data extraction

Based on the Cochrane Consumers and Communication Review Group’s data extraction template [55], sociodemographic, methodological, results, and quality appraisal details were systematically recorded in an Excel spreadsheet. Prior to implementation, the data extraction spreadsheet was piloted and refined [44].

Included articles were independently appraised by two reviewers. Data were extracted by a primary reviewer and checked for accuracy and clarity by a second reviewer. As necessary, a third reviewer was called upon to also appraise the article and contribute to the consensus decision. Authors of the original research were contacted for additional information or to clarify aspects of the method where necessary. In making these contacts, we emailed multiple authors from the same article and followed up 2 weeks later if no response was received.

Where data for the same participants were reported across multiple studies, subject numbers, demographics, and outcomes were compared for discrepancies. If there was uncertainty about the similarity of the study participants and results, authors of the original research were contacted for clarification. Where the same subjects were included in multiple studies, reference was made to all the studies but data were treated as a single source.

### Data summary and reporting

Review findings were reported as a narrative given that the small number of studies were heterogeneous in terms of their design, outcome measures, and subjects. For some topics (i.e., mortality, ipsilateral reamputation) the literature included a larger number of studies reporting the same outcome, at the same time points, in similar populations that allowed for meta-analysis. In these cases, meta-analyses were conducted using proprietary software (StatsDirect Ltd, Cheshire, UK) and a random-effects model used given the assumption that treatment effects varied across studies [56]. Where possible, proportional meta-analyses were undertaken for each time point to obtain point estimates and 95% confidence intervals (95%CI) for the PFA and TTA cohorts. Similarly, relative risk (RR) meta-analyses were undertaken to compare the RR between PFA and TTA cohorts. Heterogeneity of results between studies was quantified using the *I* ^2^ statistic and explored using the findings of the risk of bias assessment. Common issues with internal and external validity (e.g., degree to which the PFA and TTA cohorts are similar) were discussed with a specific focus on limitations that lead to imprecision, indirectness, inconsistency, and publication bias [57]. The extent to which these issues impact the results was discussed and lead to an understanding about which studies engender the most confidence in the results and why. Findings were first reported for a PFA sample, irrespective of the level of amputation. Where possible, results were also reported with a breakdown by level of PFA and compared to the outcomes of people with TTA.

## Results

### Study selection

**Fig. 1:**
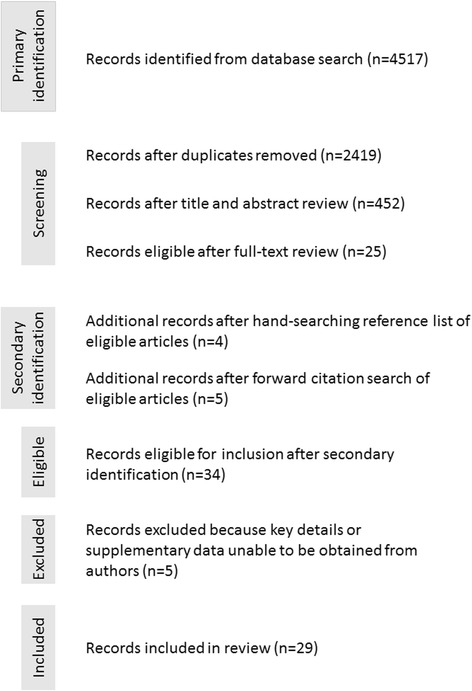
PRISMA flowchart

As summarized in the PRISMA flowchart, the search yielded 4517 articles (Fig. 1). After duplicates were removed, 2419 articles were vetted against the inclusion criteria based on the title and abstract. A total of 452 full-text articles were reviewed. Of these, 25 articles met the inclusion criteria. The references of these 25 articles were hand-searched resulting in an additional 4 articles that met the inclusion criteria. Forward citation searching identified additional five articles that met the inclusion criteria. Of the 34 articles that met the inclusion criteria, 5 were excluded because we were unable to confirm eligibility after efforts to contact authors (2 articles) or obtain the necessary data (e.g., data reported as a figure without supporting numerical data). In total, 29 articles were included in the review.

### Study characteristics

The overwhelming majority of included studies reported outcomes for populations in the USA with isolated studies in similarly developed countries [36, 38, 58–64].

Most of the included studies reported on mortality [17, 18, 28, 59–63, 65–74] (Table 2). There were a small number of investigations reporting outcomes related to functional ability [35, 75], mobility [17, 33, 34, 76], QoL [35, 36, 38], ipsilateral reamputation [27, 28, 58, 64–66, 70], and wound healing and complications [18, 64, 74, 77]. No studies that met the inclusion criteria reported on pain, participation, or psychosocial outcomes.

**Table 2:** Summary of study design and outcomes of included studies

Author	Study design	Outcomes					
		Wound healing	Ipsilateral reamputation	Quality of life	Functional ability	Mobility	Proportionate mortality
Andrews et al. [77]	Cohort—retrospective	Healed at 3 and 12 months					
Boutoille et al. [38]	Case control			Medical Outcome Study Short Form 36 (MOS SF-36)			
Brown et al. [17]	Cohort—retrospective					Volpicelli ambulatory scale	Mortality at 1, 3, 5 years
Czerniecki et al. [76]	Cohort—prospective					Locomotor capabilities index 5	
Czerniecki et al. [34]	Cohort—prospective					Locomotor capabilities index 5	
Dillingham and Pezzin [66]	Cohort—retrospective		Reamputation at 1 year				Mortality at 1 year
Dillingham et al. [28]	Cohort—retrospective						Mortality at 1 year
Evans et al. [18]	Cohort—retrospective	Healed at 1 year	Reamputation at 1 year				Mortality at 2 years
Glaser et al. [65]	Cohort—retrospective		Reamputation at 1 and 5 years				Mortality at 1, 3, 5 years
Griffin et al. [59]	Cohort—retrospective						Mortality at 30 days and 1 year
Hambleton et al. [63]	Case control						Mortality at 3 and 6 months and 1 and 5 years
Izumi et al. [27, 67]	Cohort—retrospective		Reamputation at 1, 3, 5 years				Mortality at 10 months and 5 years
Jindeel and Narahara [68]	Cohort—retrospective						Mortality at 1 and 5 years
Jones and Marshall [69]	Cohort—retrospective						Mortality at 3 and 5 years
Kono and Muder [70]	Cohort—retrospective		Reamputation at 6 months and 3 year				Mortality at 3 years
Kristensen et al. [61]	Cohort—retrospective						Mortality at 30 days and 1 year
Lakstein et al. [64]	Cohort—retrospective	Healed at 3 weeks	Reamputation at 4 weeks				
Marzen-Groller et al. [75]	Cohort^a^				Modified functional independence measure		
Mayfield et al. [71]	Cohort—retrospective						Mortality at 30 days and 5 years
Norvell et al. [33]	Cohort—prospective					Locomotor capabilities index 5	
Peters et al. [35]	Cohort—retrospective			Sickness impact profile	Sickness impact profile		
Quigley et al. [36]	Cross-sectional			Medical Outcome Study Short Form 36 (MOS SF-36 version 2)			
Sandnes et al. [72]	Cohort—retrospective						Mortality at 30 days, 1 and 5 years
Sheahan et al. [73]	Cohort—retrospective						Mortality at 30 days, 1 and 5 years
Stone et al. [74]	Cohort—retrospective	Healed at 3 months					Mortality at 60 days, 1, 3 and 5 years
Vamos et al. [60]	Cohort—retrospective						Mortality at 30 days and 1 year
Winell et al. [62]	Cross-sectional						Mortality at 2 years
Younger et al. [58]	Case control		Reamputation at 1 year				

^a^Unclear if this study is retrospective or prospective

Given the different topics included in the review, it was perhaps not surprising that study designs varied (Table 2). For example, most studies of mortality were retrospective cohort studies of large national [28, 60, 66] or state [71, 72] databases (Additional file 2: Table S6) that resulted in very large participant numbers (*n* > 1000, Table 3). By contrast, studies on QoL used case control [35, 38] or cross-sectional [36] designs (Table 2) as appropriate to the aims and recruited small samples (*n* < 30) when stratified by level of amputation (Table 3).

**Table 3:** Summary demographic characteristics of included studies

Author	Partial Foot Amputation				Transtibial Amputation				A	B	C	D	E	F	G	H	I	J	K	L	M	N	O	P	Q	R	S	T	U	V	W
	(n)	DM (%)	Male (%)	Age M(SD)	(n)	DM (%)	Male (%)	Age, M(SD)																							
Andrews et al. [77]	307	79	70	65.5(14.8)							X			X			X				X	X									
Boutoille et al. [382]	19	100		68 (median)^	6	100		68 (median)^						X		X	X														X
Brown et al. [173]	64^	100		57˭	18	100		59(12)						X		X				X							X	X			
Czerniecki et al. [76]	27	86	≈96^˭	≈64^˭	39	86	≈96^	≈64^	X	X	X	X				X	X		X	X	X			X	X	X	X	X			X
Czerniecki et al. [34]	27	100		63(8)	52	85		62(9)	X	X	X	X				X	X		X	X	X			X	X	X	X	X			X
Dillingham and Pezzin [66]	379	70^	49^	75^	949	70^	49^	75^	X	X										X										X	
Dillingham et al. [28]	1451	83	52^	74^	950	79	52^	74^	X											X										X	
Evans et al. [18]	88	100	57	70	25	100	76	69									X	X			X		X		X		X		X		X
Glaser et al. [65]	1140	74	67	67(14)					X								X				X						X				
Griffin et al. [59]	63	68	87	69					X		X						X				X		X	X		X	X			X	
Hambleton et al. [63]	123	100	45^	67(13)	47	100	45^э^	76(10)^ø^	X			X			X	X	X	X			X	X			X						X
Izumi et al. [27, 67]	213	100	70˭	52˭					X		X												X	X	X	X	X				
Jindeel and Narahara [68]	594	93	75^	≈55^α^					X								X														
Jones and Marshall [69]	29	100	100	71 (7)	30	100	100	71(7)						X	X			X			X		X	X	X		X				
Kono and Muder [70]	116	80	99	67(11)							X	X	X				X	X				X	X			X	X		X	X	X
Kristensen et al. [61]	15	53	80	74(15)	31	39	58	74(13)		X										X									X	X	
Lakstein et al. [64]	35	100	66	66.6(10.2)									X																		X
Marzen-Groller et al. [75]	13				19																										
Mayfield et al. [71]	2234	61^	99^	66^	1381	61^	99^	66^	X	X							X								X		X				
Norvell et al. [33]	27	100	100	63(8)	52^b^	85	88	62(9)	X	X	X	X				X			X	X	X			X	X	X	X				X
Peters et al. [35]	26	100	77^	57(10)^	8	100	77^	57(10)^						X		X	X					X	X								
Quigley et al. [36]	10	80	70	63(10)	23	70	60	68(10)									X	X									X				X
Sandnes et al. [72]	4434	66	61	66(16)	5298	63	61.9	68(16)									X			X					X		X				X
Sheahan et al. [73]	670	92	70								X						X				X		X				X				
Stone et al. [74]	74	100	87	64 (No SD)							X				X												X		X		
Vamos et al. [60]	5487*	40^	59˭^ψ^	66˭^ψ^						X							X														
Winell et al. [62]	706	100															X														
Younger et al. [58]	21	100	81	60.4(11.8)	21	100	81	56.3(10.9)			X		X	X			X										X				

A: Race/Ethnicity; B: Socio-demographic details; C: Smoking/Tobacco Use; D: Other Lifestyle Behaviors; E: Exams/Tests; F: HbA1c; G: Other Biomarkers; H: BMI; I: Diabetes; J: Sensation; K: Mental Health; L: Comorbidities; M: Blood Pressure; N: Other Blood Flow/Pressure Issues; O: CAD; P: Stroke/CVA; Q: Other Cardiovascular Issues; R: Revascularization; S: Renal Issues; T: Mobility; U: Re-amputation; V: Amputation Surgery; W: Other; *DM* Diabetes Mellitus, *M(SD)* Mean (Standard Deviation), *TMA* Transmetatarsal Amputation; Sociodemographic details include education level, relationship status, employment status, residential status, geographic region, economic status, Modified Social Support Score; Other lifestyle behaviors include alcohol use and malnutrition; Exams/Tests include physical exams, lab tests and cultures; Other biomarkers include GHb and hyperlipidemia; Mental health includes major depressive disorders and post-traumatic stress disorder; *BMI* Body Mass Index; Sensation includes neuropathy, impaired protective sensation, Vibration Perception Threshold; Comorbidities include the Charlson Comorbidity Index, frequency of comorbidities and the Anesthesiology Association of America Physical Status scale; Other blood flow/pressure issues include missing pulses, ankle brachial index, Doppler for ankle blood pressure; *CAD* Coronary Artery Disease, *CVA* Cerebrovascular Accident; Other Cardiovascular problems include cardiovascular disease, myocardial infarct, heart attack, peripheral arterial disease, peripheral vascular disease and chronic obstructive pulmonary disease; Renal issues include nephropathy, renal dialysis, chronic renal failure, renal insufficiency and end stage renal disease; Mobility include the Locomotor Capability Index-5 and Volpicelli Ambulatory Scale; Amputation surgery includes urgency of surgery, cause/level of amputation, age at first amputation, operation time, length of stay, number of amputation related admissions in 12 months post-index amputation, wound healing, post-op infection and post-op antibiotic use; Other includes immunosuppressant use, joint replacement, Charcot collapse, footwear type, perceived premorbid health, retinopathy, ulcer and traumatic brain injury. ^based on lower limb sample as a whole. *in year 2005. ^b^with 10 drop outs over 12 months. ^α^stratified by sex. ^ψ^in most recent year reported (2005/6). ^э^based on case subjects for whole lower limb sample; ^ø^based on ‘major’ group including transtibial/transfemoral amputation. ˭weighted to summarize data presented by discrete cohorts (e.g., different amputation levels)

By virtue of the inclusion criteria, all studies reported data for people with dysvascular PFA. Most studies reported data for a single group that included people with different levels of PFA [18, 27, 28, 35, 36, 38, 60–63, 65, 68, 70, 72, 73], often described by collective nouns such as *midfoot* or *minor* amputation. Some data were reported for discrete levels of PFA including toe [27, 28, 59, 64, 67, 69, 71], TMA [33–35, 58, 71, 74–76, 78], ray [67], transtarsal [78], or partial/total calcanectomy [78]. A number of studies also included a cohort with TTA [28, 33–36, 38, 61, 63, 69, 71, 72, 75, 76, 78].

Given the population of interest in this review (i.e., people with dysvascular PFA or TTA), it was not surprising that studies included cohorts that were typically older and male and had large proportions with diabetes among other comorbidities (Table 2).

### Quality appraisal/risk of bias

Based on the McMaster Critical Review Form, there were a number of recurrent issues affecting both the internal and external validity of studies included in the review. While a detailed analysis of included studies has been presented as part of the appendices (Additional file 3) and contextualized as part of the results narratives, a summary of common issues has been included below.

Given the inclusion of people with dysvascular amputation, it was not surprising that a large proportion of participants were older, male, and diabetic (Table 3). Unfortunately, there have been a disproportionate number of studies that only included males [33, 34, 69, 70] or people with diabetes [17, 18, 27, 34, 35, 38, 58, 62–64, 67, 69, 74]. While most studies reported these salient details, more detailed information about race, sociodemographic factors, or the presence of common comorbidities were reported in an ad hoc way and as such, it was not possible to make a detailed assessment of the degree to which people included in research represented the broader population of people facing PFA or TTA (Table 3).

There were a number of sources of bias common to studies included in the review (Additional file 3). Contamination and co-intervention were common, particularly the use of treatments as an adjunct to surgery (e.g., vacuum-assisted closure therapy) or to manage comorbidities (e.g., renal impairment), which complicated studies of wound healing, reamputation, and mortality rates. No studies justified the sample size (Additional file 3). In comparison to studies of mortality or reamputation rates, investigations of mobility, QoL, or functional ability tended to include small subject numbers (Additional file 2: Table S3, S4, S5), which is not atypical of amputation research. While most studies used reliable and valid outcome measures, studies of wound healing did not operationally define what constituted a healed wound (Additional file 2: Table S1).

### Wound healing and complications

Four retrospective cohort studies met the inclusion criteria [18, 64, 74, 77] and reported the proportion that healed following PFA at discrete time points (Table 2). Studies included people with toe [64], TMA [74], or different levels of PFA in a single cohort [18, 77]. None were designed to compare between different levels of PFA (Additional file 2: Table S1). While one study [18] included a PFA and TTA cohort, the rate of wound healing was not reported for the TTA cohort; instead it was implied that 100% of people with TTA healed as none progressed to transfemoral amputation [18] (Additional file 2: Table S1).

While participants in these studies were fairly representative in terms of their age and sex distribution, most studies only included people with diabetes [18, 64, 74] (Table 3). Detailed information about the presence of comorbidities (e.g., renal impairment) were reported in an ad hoc fashion, which made it impossible to assess the extent to which these comorbidities influenced healing after amputation (Additional file 2: Table S1).

No studies operationally defined a healed wound (Additional file 2: Table S1). Definitions such as “*adequately healed*” [74] or “*well-healed*” [64] typify the imprecision that likely contributed to the heterogeneity of results between studies.

Although all studies described the proportion of the sample that healed following PFA, there were few studies reporting the outcome at any given time point. The proportion of the sample that healed were reported for 3 weeks [64], 3 months [74, 77], and 1 year [18, 77] after PFA. In one study [77], the proportion that healed at 1 year was calculated by combining the proportion of the sample that healed at 3 months and those that healed at some stage between 3 and 12 months.

Acknowledging the limited literature available, results suggested that about 50% of PFAs healed at 3 months (43–61%) [74, 77] and about 75% healed by 1 year (70–88%) after amputation [18, 74]. There was considerable uncertainty about these estimates given that they were based on isolated studies, each with their own biases (Additional file 3: Table S1). For example, the proportion of the sample that healed at 3 and 12 months may be artificially low in the study by Stone et al. [74] given that nearly all required a previous distal amputation and/or debridement to control sepsis and were chronically unwell as evidenced by the high proportion (26%) with end-stage renal disease and contralateral lower limb amputation (16%).

Given such uncertainty in the evidence, the proportion that healed at 3 weeks were not reported given that those most likely to experience complications (e.g., those with extensive cellulitis or ischemia requiring revascularization) were excluded, therefore biasing the result to the extent that outcomes were inconsistent with other studies [64] (Additional file 3: Table S1).

Wound healing after PFA was complicated in a high proportion of cases [18, 74]. For example, Evans et al. [18] reported a perioperative complication rate of 42% in a PFA cohort (time not defined), which reflected the proportion of the sample that experienced dehiscence/necrosis or ipsilateral reamputation (Additional file 2: Table S1). The proportion of the sample that experienced perioperative complications was reportedly lower in the TTA cohort (28%), reflecting slightly different complications including dehiscence/necrosis, bleeding, and local wound revision. Although none operationally defined the outcome, it was likely that the definition of a complication varied across studies as did the use of parallel treatment (e.g., hyperbaric oxygen therapy) that would also likely have affected the proportion that healed after surgery (Additional file 3: Table S1).

### Ipsilateral reamputation

Seven retrospective cohort studies met the inclusion criteria, Table 2 [27, 28, 58, 64–66, 70]. Two [28, 66] used the same population and were considered as a single data source. While all studies described the proportion of the sample that had undergone ipsilateral reamputation at a specific time point following PFA, the time points were not the same across studies: 4 weeks [64], 6 months [70], 1 year [27, 28, 58, 65], 3 years [27, 70], and 5 years [27, 65]. While there were enough studies to calculate a point estimate and 95% confidence interval for ipsilateral reamputation at 1 year, there were too few studies at the other time points; hence, a narrative approach was used to describe proportionate ipsilateral reamputation for 3 and 5 years after PFA.

**Fig. 2:**
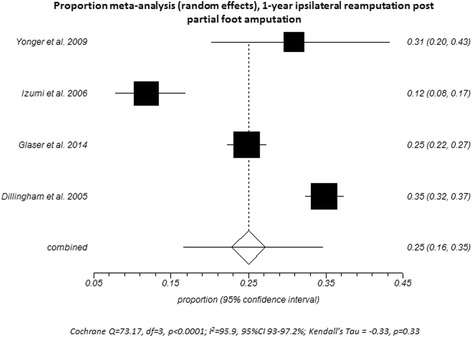
Proportional meta-analysis (*random effects*) for ipsilateral reamputation at 1 year after PFA

Notwithstanding the limited literature available, results indicate that 25% (95%CI, 16.47–34.64) of people required ipsilateral reamputation within 1 year of their PFA (Fig. 2). Two studies reported data for multiple time points [27, 65] highlighting that proportionate ipsilateral reamputation increased over time (Additional file 2: Table S2). By way of example, Izumi et al. [27] reported that proportional ipsilateral reamputation increased from 1 year (25%) to 3 years (39%) and 5 years (49%) after PFA (Additional file 2: Table S2). While this increasing proportion with ipsilateral reamputation over time was consistent across studies, the rates at 3 years (49%) [70] and 5 years (34%) [65] were very different, highlighting the heterogeneity of results between studies (Additional file 2: Table S2).

There was considerable uncertainty about proportionate ipsilateral reamputation given the limited number of studies and heterogeneity of the results between studies. The representativeness of the cohorts was of concern in more than half of the included studies (Additional file 3: Table S2). For example, Izumi et al. [27] likely under-represented the true proportion that progressed to ipsilateral reamputation given that the cohorts were relatively young (Table 3) where the long-term effects of diabetes are unlikely to have impacted the outcome (Additional file 3: Table S2). Issues with counting of reamputations were common as exemplified by studies that did not count: subsequent amputations within a 2-week period of the index (first ever) amputation [27], reamputation in people who died within the first month [70], or cases where more proximal amputation occurred within the same admission [28] (Additional file 3: Table 2). By contrast, other studies likely over-represented the rate of proportional ipsilateral reamputation given cohorts included older people [28, 66], many with prior amputation [58], and serious comorbid conditions such as coronary heart disease [70] (Additional file 3: Table S2).

There were too few studies reporting data for people with toe [27, 28, 64], ray [27], or TMA [58] at any time point to produce meaningful estimates of proportionate ipsilateral reamputation for different levels of PFA. Only one of these studies, Izumi et al. [27] was designed to compare between levels of PFA, and while we have raised uncertainty about the representativeness of the proportionate reamputation rate reported in this study, the comparison between different levels of PFA was fair because the age bias affected the different PFA groups equally. As such, the investigation by Izumi et al. [27] was best designed to inform our understanding, highlighting that there were no difference in the proportion of people who progressed to ipsilateral reamputation at 1, 3, or 5 years when stratified by toe, ray, or midfoot (i.e., TMA, Lisfranc, Chopart) amputation (Additional file 2: Table S2).

Only one study [28] included a PFA and TTA cohort; unfortunately, the study was not designed to compare between levels of amputation and as such, only descriptive data were available. Results suggest that there was little difference between the proportion of people with PFA (35%) and TTA (33%) that progressed to ipsilateral reamputation within 1 year [28]. However, it was likely that the true proportion of the PFA cohort that progressed to ipsilateral reamputation was under-estimated because people with toe amputation(s) were excluded [28] (Additional file 2: Table S2).

### Quality of life

Three studies reported QoL outcomes including two case control studies [35, 38] and one cross-sectional study [36] (Table 2). All studies used generic measures of QoL including the Medical Outcome Study Short Form 36 (SF-36) [36, 38] or the Sickness Impact Profile (SIP) [35].

Participants were broadly representative of the dysvascular amputee population given that studies included older adults with amputation due to peripheral vascular disease following many years of living with diabetes (Table 3). Different comorbidities were reported in all three investigations to characterize the cohorts (Table 3). The total number of subjects included in comparison groups were small (*n* < 30), especially considering that groups included varying levels of PFA (Table 3).

The two case control studies [35, 38] were designed to compare QoL in subjects with amputation to those without; these studies were not designed to compare QoL between groups with different levels of amputation (Additional file 2: Table S3). One cross-sectional study [36] was designed specifically to compare QoL in cohorts with PFA and TTA and used a multivariate analysis of covariance (MANCOVA) to control for the confounding influence of age, time living with diabetes, and the presence of diabetic complications (i.e., retinopathy, neuropathy, nephropathy). While the study by Quigley et al. [36] was limited by the small PFA group (*n* = 10) and included people with different levels of PFA in that group, this investigation provided the best available evidence given that it was designed to compare different levels of amputation and controlled for covariates known to influence QoL (Additional file 3: Table S3).

Overall, the very limited evidence suggests that QoL was comparable in people with PFA and TTA. No studies have compared QoL in people with different levels of PFA. Factors that significantly influence QoL included advancing age, longer time living with diabetes, and the presence of retinopathy [36]. Given that amputation at either the PFA or TTA level did not have a significant influence on QoL [36], it seems reasonable to suggest that QoL is unlikely to differ between levels of PFA.

### Functional ability

While two studies on functional ability met the inclusion criteria [35, 75], one was incomprehensible [75] (Additional file 3: Table S4). It seemed that the study by Marzen-Groller et al. [75] modified the Functional Independence Measure (FIM) to focus on seven activities relating to transfers, bed mobility, sit-to-stand and distance walked, presumably each with their own score. While three of these measures were reported in the results, it was not clear whether the results described a change score, an average score, or observations at one time point (Additional file 3: Table S4). While these issues are by no means exhaustive, they represent a critical limitation, that is, it was not clear what was measured and as such, the study cannot inform our understanding of functional ability after PFA.

The second of these investigations [35] used the SIP to compare functional ability and activity restriction in three groups with diabetes and either PFA, TTA and/or transfemoral amputation, or no amputation [35]. The results indicated that the total SIP scores were comparable in the PFA and no amputation groups, despite the differences in the physical dimension subscale scores (Additional file 2: Table S4). This study was not designed to compare between levels of PFA or between cohorts with PFA and TTA given the heterogeneous group of “major” amputations that included people with TTA and transfemoral amputation [35]. The ability to identify small differences between these groups was limited because results were highly variable and groups were not matched to control for the confounding influence of systemic disease (e.g., duration of diabetes) nor were these differences controlled for statistically (Additional file 3: Table S4). It was also difficult to generalize these results given that demographic and health characteristics were not reported by amputation level (Additional file 3: Table S4). Given these limitations, it was not possible to vest confidence in these results without other studies to corroborate these findings.

### Mobility

Four cohort studies on mobility met the inclusion criteria, Table 2 [17, 33, 34, 76]. Given that the same participants were included in two of the studies [33, 34] and that a subset of this sample was used in the third study [76], these studies were considered as a single source (Additional file 2: Table S5).

There were substantive methodological concerns with the one study that used the Volpicelli ambulatory scale [17]. The Volpicelli ambulatory scale was inappropriate for use in this unilateral population because it was designed for use in people with bilateral limb loss [79]. It is likely that some participants with unilateral amputation experienced a ceiling effect with the use of this measure (Additional file 3: Table S5). The results were reported using means and standard deviations, which was also inappropriate given the measure’s ordinal scale and rendered the mutually exclusive categories of the original measure meaningless (Additional file 3: Table S5). The small differences reported between groups are likely to be spurious, and the results should be interpreted with caution (Additional file 3: Table S5). Due to these concerns, the study was unsuitable to inform our understanding of mobility.

Participants in the remaining three studies [33, 34, 76] were broadly representative of the dysvascular amputee population as evidenced by their average age, cause of amputation, and presence of common comorbidities (Table 3). However, all participants were male and a large proportion (>85%) had diabetes [33, 34, 76]. These studies included cohorts with TTA and TMA [33, 34, 76].

The Locomotor Capabilities Index-5 (LCI-5) was used to assess mobility over time (i.e., premorbid mobility recalled at 6 weeks post-amputation and mobility at 4, 6, and 12 months post-amputation) and between cohorts with TMA, TTA, and transfemoral amputation [33, 34, 76]. While these studies [33, 34, 76] were generally methodologically strong, recall bias may have influenced the ability of participants to rate their premorbid mobility 6 weeks after amputation (Additional file 3: Table S5). However, this issue does not affect the conclusion that from 6 weeks post-amputation, mobility continued to improve at each successive time point until the last measure at 12 months for both the TMA and TTA cohorts [34]. While these studies were not designed to compare mobility at 6 weeks or 4 months post-amputation, descriptive data suggests that mobility was better for the TMA cohort than for the TTA cohort at these time points [34] (Additional file 2: Table 5). However, at 12 months post-amputation self-reported mobility seemed to be very similar for people with TMA and TTA [33, 34] (Additional file 2: Table S5). At the 12-month follow-up, only one-third of people with TMA or TTA returned to their premorbid level of mobility [33].

### Mortality

Of the 22 articles that reported mortality outcomes and met the inclusion criteria, we were unable to obtain complete data [80, 81] or verify critical details to confirm inclusion [82, 83] from the authors of four articles; hence, 18 studies were reviewed (Additional file 2: Table S6). While most studies were retrospective cohort studies [17, 18, 28, 59–61, 65–74], there was one cross-sectional [62] and one case control [63] study (Table 2). Two studies [28, 66] examined the same population and were therefore treated as a single data source. A number of studies included very large samples [28, 60, 62, 65, 66, 68, 71, 72] typical of national- or state-based population studies, which added considerably to the power with which conclusions about mortality could be made (Table 2).

While our protocol specified inclusion of studies reporting a mortality rate, the term was imprecisely used among studies. Only one study [63] reported a true mortality rate (i.e., age-standardized all-cause mortality rate per 1000 person-years). However, all included studies reported all-cause proportionate mortality (or survival from which mortality was calculated) and included additional information about the time point at which it was measured (Additional file 2: Table S6).

Proportionate mortality was determined either by counting the proportion of the sample that died over time [6, 18, 28, 59, 61, 68–70] or by using a Kaplan-Meier analysis [17, 62, 63, 65, 67, 71, 73] (Additional file 2: Table S6). In one study, it was not clear which of these two approaches was used to generate the proportionate mortality reported [72] (Additional file 2: Table S6). Most commonly, proportionate mortality was reported for a perioperative period of 30 days [59–61, 71–73] or for 1 year [17, 28, 60, 61, 63, 65, 68, 72–74], 3 years [17, 65, 69, 70, 74], or 5 years [63, 65, 67–69, 71–74] after amputation, acknowledging that not all studies reported proportionate mortality at each time point. There were isolated data for other time points including 3, 6, and 10 months or 2 years post-amputation [18, 62, 63, 67, 74].

A number of studies reported proportionate mortality for a PFA group without breakdown by level of amputation [18, 60–63, 68, 70, 73]. A few studies included more refined data for cohorts that included multiple levels of PFA (e.g., toe/TMA) [28, 65, 72] or discrete levels including toe(s) [28, 59, 67, 69, 71], ray [67], TMA [17, 71, 74], Chopart [17], or calcanectomy [84]. A number of these studies also included a TTA cohort [28, 61, 63, 69, 71, 72, 84].

Given the number of studies that reported the same outcome at standard time points in similar cohorts with dysvascular PFA, we conducted a series of proportional meta-analyses to integrate the results from these independent studies and provide point estimates of proportionate mortality. All studies were included in the meta-analyses in keeping with the view that there is a common truth behind similar studies and that preserving information about the heterogeneity of results between studies is important to explain underlying causes of variation and uncertainty in the point estimates reported [85].

**Fig. 3:**
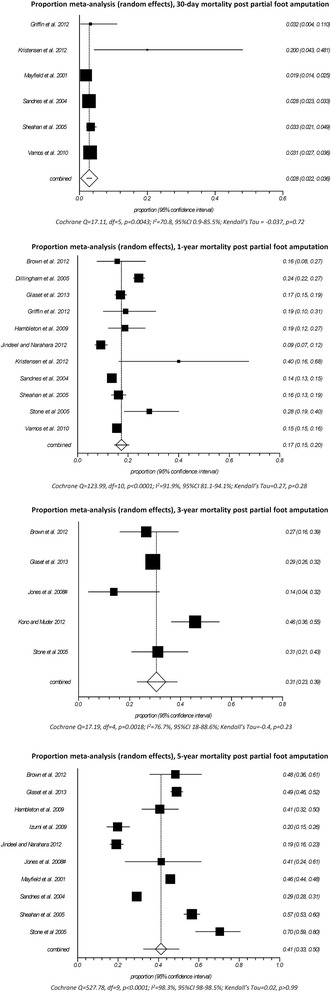
Proportional meta-analysis (*random effects*) for mortality at 30 days, 1, 3, and 5 years after PFA

Following PFA, proportionate mortality increased from 30 days (2.8%, 95%CI 2.2–3.6) to 1 year (17.3%, 95%CI 14.6–20.2), 3 years (30.6%, 95%CI 23.0–38.7), and 5 years (41.2%, 95%CI 32.6–50.1) (Fig. 3). There was considerable heterogeneity in the results between studies (*I* ^2^ > 70%, *p* < 0.01) and while this did not detract from the precision of the point estimate at 30 days, estimates of proportionate morality became less precise over time (Fig. 3). Fortunately, studies that most contributed to the heterogeneity received less weighting in the random-effects meta-analysis and thereby had the least influence on the point estimates.

Given that measures of proportionate mortality do not control for variations in the sample characteristics (e.g., older age), this was a notable source of variation in the results (Additional file 2: Table S6). For example, the study by Kristensen et al. [61] included a sample that was old (75.8 ± 11.4 years), chronically unwell (20% had 4–5 comorbidities, 10% had American Society of Anesthesiologists (ASA) scores ≥4 indicative of a systemic disease that is life threatening or people who are not expected to survive), and included a large proportion (18%) with prior amputation (Additional file 2: Table S6). As such, it was not surprising that proportionate mortality was multiple times greater compared to studies with more representative samples (Fig. 3). Proportionate mortality was also greater than might be expected in other studies where the samples were similarly unrepresentative [17, 70, 73, 74]. By contrast, proportionate mortality was low in two studies [67, 68] given that only those with index amputation were included and participants were relatively young where the serious complications of diabetes might not yet have increased the risk of mortality (Additional file 3: Table S6).

Aside from sample characteristics, heterogeneity of the results between studies was also due to variation in the *usual care* provided (Additional file 3: Table S6). Variations in care are not surprising given that most studies were retrospective reviews of national- or state-wide datasets that included outcomes from multiple healthcare providers over many years. While issues with co-intervention and contamination were typically not addressed (Additional file 3: Table S6), some investigations described aspects of care that likely vary across studies. For example, Evans et al. [18] described routine tendon rebalancing, hyperbaric oxygen therapy (24% of the sample used this treatment), and multiple debridement procedures (average two per person) following PFA (Additional file 3: Table S6). In other studies, revascularization procedures either prior to, or in parallel with, amputation were reported [59, 65, 74], as were lengthy periods of non-weight bearing [74] and the use of vacuum-assisted closure therapy [59, 65] (Additional file 3: Table S6). Considering that many people facing PFA also have serious comorbidities (e.g., renal insufficiency, congestive heart failure), treatments in addition to those directly related to the amputation are likely to have influenced mortality.

When considered with respect to level of PFA, there were too few studies (≤3) reporting proportionate mortality at any time point to warrant meta-analysis, particularly given that these studies [17, 67, 74] have already been shown to influence the heterogeneity of results between studies and, without other investigations with which to compare the outcome, it was difficult to vest confidence in the result of the meta-analysis of isolated studies. Of the studies that reported proportionate mortality for different levels of PFA [17, 28, 67, 71], only one was designed to compare between levels [67] and, as such, this study was best designed to inform our understanding. Izumi et al. [67] found no statistically significant difference in proportionate mortality between groups with toe, metatarsal ray resection, or *midfoot* amputation (i.e., cohort included TMA, Lisfranc and Chopart amputations) at 10 months (toe 6.6%, ray 4.4%, midfoot 10.5%) and 5 years (toe 26.2%, ray 15.8%, midfoot 21.0%) (Additional file 2: Table S6).

Following TTA, proportionate mortality increased from 30 days (7.8%, 95%CI 5.2–10.8) to 1 year (34.2%, 95%CI 22.5–46.9) and 5 years (54.6%, 95%CI 42.9–66.1). There were insufficient studies reporting 3-year proportionate mortality to warrant a meta-analysis, particularly given the small number of subjects in these studies [17, 69] (Additional file 2: Table S6).

**Fig. 4:**
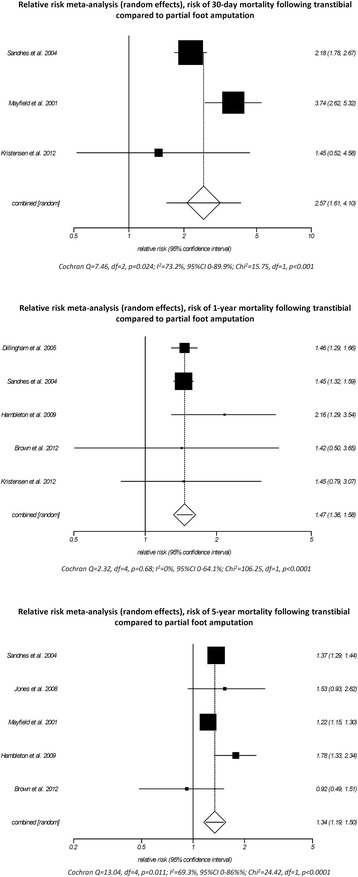
Relative risk meta-analysis (*random effects*) comparing mortality at 30 days, 1 and 5 years after PFA and TTA

In comparison to people with PFA, those with TTA had a significantly greater RR of dying at 30 days (RR 2.6, 95%CI 1.6–4.1, *p* < 0.001), 1 year (RR 1.5, 95%CI 1.4–1.6, *p* < 0.001), and 5 years (RR 1.3, 95%CI 1.2–1.5, *p* < 0.001, Fig. 4). It is important to note that the RR of mortality was disproportionately high in the perioperative period compared to 1 and 5 years after amputation (Fig. 4). Two studies [71, 72] that most influenced the RR meta-analysis at 30 days post-amputation both operationally defined the highest amputation level as the index amputation (Additional file 3: Table S6). If we assume that the 15% of people who required multiple amputations in the same admission [71] are also those with the worst health and therefore at greatest risk of dying, and we categorize them by the highest level of amputation, it artificially inflates the RR of perioperative mortality toward TTA (Additional file 3: Table S6).

While the RR of perioperative mortality may be artificially inflated toward those requiring TTA, comorbid-specific Kaplan-Meier analyses [71] highlighted that the very high mortality in the first year after amputation were associated with renal disease, congestive heart failure, and cerebrovascular disease (Additional file 3: Table S6). Unfortunately, demographic and comorbid conditions were not reported by cohorts with PFA and TTA [71], which made it impossible to assess the extent to which the presence of these conditions differed between cohorts and contributed to the higher RR of perioperative mortality for people with TTA compared to those with PFA (Additional file 3: Table S6).

Given an understanding of the leading causes of death after amputation [63, 71], the increased RR of dying for those with TTA may not be due to the amputation per se given that, in all likelihood, people with TTA have more advanced systemic disease that both increased the RR of dying and necessitated more proximal amputation.

The degree to which heterogeneity of the results varied between studies depended on the time point (Fig. 4). Of particular interest was the heterogeneity between results for 30 days post-amputation (*I* ^2^ = 73.2%, *p* = 0.024, Fig. 4) given the similarity of two of these studies [71, 72]; both include very similar populations based on the demographic and comorbid conditions reported (Table 3) and operationally defined the index amputation as the highest amputation level (Additional file 3: Table S6). Unfortunately, one of these studies [71] did not report demographic and comorbid conditions by cohorts with PFA and TTA, making it impossible to assess the extent to which the sample characteristics influenced the heterogeneity of results between studies (Additional file 2: Table S6). While issues with the representativeness of the sample described by Kristensen et al. [61] have already been discussed, these were of little consequence given that the other studies reporting 30-day proportionate mortality [71, 72] were less heterogeneous and thereby dominated the point estimate (Fig. 4).

## Discussion

The purpose of the review was to describe a comprehensive range of outcomes following dysvascular PFA and to compare these between levels of PFA and TTA.

Aside from mortality, there was limited evidence regarding outcomes of dysvascular PFA, particularly how outcomes differed between levels of PFA and TTA. The available evidence suggests that

- *Wound healing and complications*: about 50% of PFAs healed at 3 months and about 75% healed by 1 year. Complications such as dehiscence and necrosis affected a large proportion of people following PFA, and these complications may affect a smaller proportion following TTA.
- *Ipsilateral reamputation*: about 25% of people with PFA required an ipsilateral reamputation within 1 year. While this proportion increased over time, there was uncertainty about the rate of ipsilateral reamputation at later time points. Given the limited evidence, there was also uncertainty that the rates of ipsilateral reamputation were comparable between levels of PFA and TTA.
- *QoL*: QoL may be similar in people with PFA and TTA. While no studies have compared QoL between levels of PFA, research suggests that older age, time with diabetes, and the presence of diabetic complications had a significant influence on QoL, where amputation level did not.
- *Functional ability*: there was uncertainty about the outcomes given just one study suggests functional ability may be similar in people with PFA and no amputation. No studies compared functional ability between levels of PFA and TTA.
- *Mobility:* for most people, mobility declined following PFA. Only one-third of people with TMA returned to their premorbid mobility level 1 year after amputation. Mobility seemed comparable in people with TMA and TTA; acknowledging that in the first months following amputation, mobility may be better for people with TMA. No studies have compared mobility between levels of PFA or reported mobility outcomes for different levels of PFA (only TMA).
- *Mortality:* about 17% of people died within 1 year of their PFA, that proportion increased over time such that by 5 years about 40% of people died. While there was uncertainty that mortality outcomes were the same in people with different levels of PFA, people with TTA had a greater risk of dying. The increased risk of death in people following TTA may not be due to the amputation per se, but may simply relect more advanced systemic disease that also required more proximal amputation.

While no studies reporting participation, pain or psychosocial outcomes included discrete groups with PFA, and therefore did not meet the inclusion criteria, there exists a broader body of literature that must be acknowledged. Our systematic search of the literature identified investigations of psychosocial outcomes that included cohorts with different levels and causes of lower limb amputation focusing on body image [86–88], sexuality [89, 90], depression [91–93], psychological adjustment to amputation [94–98], and life goal adjustment [99]. A number of studies also described links between psychological features such as stress or depression and phantom limb pain [100–105]. Similarly, studies reporting participation outcomes in people with limb loss have investigated return to work [106], negotiation of environmental barriers [107], social activity participation [108, 109], and participation and autonomy [110].

Given this understanding, investigations that stratify by level of amputation are needed to more fully understand the effect of PFA and how outcomes vary between levels of PFA and TTA. Such studies could easily address many of the methodological issues highlighted in this review including operationally defining the outcome of interest (e.g., using a standardized wound classification system to measure healing), standardizing time points for measurement (e.g., perioperative mortality might always be 30 days after amputation), and stratification by level of amputation—even as a secondary outcome. As the body of literature grows, these small changes to method design will facilitate future synthesis of the evidence.

### Application of evidence to the development of shared decision-making resources

Critical appraisal and synthesis of the outcomes of PFA and how these compared between levels of PFA and TTA, provides a well-evidenced foundation for building shared decision-making resources. For example, point estimates for mortality at 1, 3, and 5 years after PFA reported in this review can be incorporated into a patient decision aid and discussion guide to help patients understand their risk of dying and inform difficult conversations at the point of amputation surgery. In this way, patients and doctors can engage in these difficult conversations with an informed understanding of the current research evidence, even acknowledging the uncertainty that exists in many outcomes. Allied health practitioners can also use evidence reported in this review to help inform discussions about the likely outcomes following PFA.

### Limitations

#### Differences between protocol and review

While developing the protocol for this review, we specified a number of outcomes of interest with reference to a rate (e.g., mortality rate). We had not appreciated that the term *rate* had different meanings in the literature. In an epidemiology context, the term *rate* has a very specific meaning: a mortality rate describes the number of people that die during a specific time period divided by the number of people at-risk of dying in that time period [111]. Given that the number of people at-risk changes over time (i.e., as people die they are no longer at-risk of dying), the mortality rate is often expressed per 1000 person-years (e.g., 50 deaths per 1000 person-years). Only one study [63] reported a true mortality rate (i.e., age-standardized all-cause mortality rate per 1000 person-years). All studies included in the review reported a proportion of the sample that died. Measures of proportion do not include information about time and, in this way, they differ from rates. Obviously, if the duration of a study was long enough, 100% of people would die. Without information about time, measures of proportionate mortality are not terribly useful to inform patients about their likelihood of dying. Ideally, measures of proportionate mortality should define the time point at which the outcome was measured (e.g., 50% of people died within 5 years of their amputation). Given this understanding, we included studies that reported a proportional mortality/wound healing/ipsilateral reamputation as long as a time point was specified and as such, it was redundant to report the outcome *time to ipsilateral reamputation* because the time point was now inherent to the definition of the ipsilateral reamputation. We hope to have been transparent in describing this change to the protocol and believe that it would have been disingenuous to exclude these studies based on the operational definition of the outcome, particularly given their importance to conversations about limb loss.

We reported outcomes related to *wound healing* after amputation rather than *wound failure* given that *wound healing* was the term used in the literature.

We also expanded the protocol to undertake a series of meta-analyses to produce point estimates for proportional mortality and ipsilateral reamputation as well as estimate the RR of mortality for those with TTA compared to PFA. At the point of designing the protocol, we had not anticipated a sufficiently large body of literature reporting the same outcomes at the same time points on any topic that make meta-analyses feasible. We believe that reporting these meta-analyses as part of the narrative review engenders greater confidence in our summary of the proportional mortality and ipsilateral reamputation results.

### Other limitations

Given the large number of outcomes included in this review, we have deliberately kept the results narratives very tight by using illustrative examples to showcase the risk of bias and the impact on the results. Detailed results of the risk of bias assessment have been included as Additional file 3, rather than in the body of the work, given the sheer size of the tables. We hope that readers can appreciate the rationale for this approach and our efforts to report all our data in its entirety for those wanting this level of detail.

During the review process, we contacted authors from 11 articles. Authors of nine articles responded, which allowed us to clarify important details necessary to determine eligibility or obtain additional data. As such, only two articles were excluded because we could not establish contact with authors.

Some may be critical that our literature search was limited to the last 15 years, particularly given that no articles on pain, participation, or psychosocial outcomes included groups with PFA and therefore did not meet the inclusion criteria. Our intention was to synthesize current research evidence given that changes in treatment practices have dramatically influenced the types of amputations being performed, cointerventions that often occur in parallel (e.g., revascularization), and the way we treat conditions such as pain or depression. As such, we felt that older literature offered little to inform our understanding of the outcomes of contemporary practice.

The quality of reporting and research methods varied markedly across investigations, and it was often difficult to draw meaningful conclusions as part of the narrative review. This is not atypical of emerging areas of research. Given that people still need to make informed decisions about their healthcare in lieu of rigorous research evidence, we hope to have guided the reader to an understanding of the best available evidence by discussing uncertainty and issues that most inform our view.

Some may be critical of our inclusion of all studies in the meta-analysis in keeping with the view that there is a common truth behind similar studies and that preserving information about the heterogeneity of results between studies is important to explain underlying causes of variation and uncertainty in the point estimates reported [85]. By exploring the heterogeneity of results using insights gleaned from our risk of bias assessment, we hope to have engendered confidence in the findings reported.

Some readers may also be critical of our decision to omit summary statistics describing the number of studies or participants from the discussion. While this may be typical, and indeed appropriate in well-developed bodies of literature, we felt it was misleading to further contextualize our discussion points based on the number of studies or participants. For example, of the four cohort studies reporting mobility outcomes [17, 33, 34, 76], we had significant concerns about use of the Volpicelli ambulatory scale [17] because it was designed for the use in people with bilateral amputation and inappropriately used in a unilateral population where some participants experience a ceiling effect. While we have explained why the study was unsuitable to inform our understanding as part of the results narrative, we are concerned that this understanding is lost when the total number of articles or participants are included as part of a summary of the key findings.

## Conclusions

This review of a comprehensive range of outcomes following dysvascular PFA and how these compare between levels of PFA and TTA highlights that evidence was limited. Notwithstanding the uncertainty that comes from small bodies of literature where the risk of bias is high, the available evidence suggests that a large proportion of people with dysvascular PFA will wait many months for wound healing and experience complications such as dehiscence or reamputation on the same limb. A large proportion of people will die within a few years following dysvascular PFA. While the risk of dying was higher for people with TTA, this may be due to advanced systemic disease that makes TTA a more appropriate amputation surgery. While outcomes such as mobility and QoL appear to be similar in people with PFA and TTA, these inferences are based on isolated studies involving small subject numbers and as such, further research is needed to be confident in these findings. No studies that reported on pain, participation, or psychosocial outcomes included discrete groups with PFA and as such, our understanding of the impact of PFA must be informed by the broader body of literature describing these outcomes for heterogeneous groups with lower limb amputation.

## Additional files

Additional file 1: PRISMA Checklist. (DOC 62 kb)
Additional file 2: Data extraction. (XLSX 146 kb)
Additional file 3: Risk of bias. (XLSX 132 kb)

## Abbreviations

ISO: International Standards Organization
LCI: Locomotor capabilities index
MeSH: National Library of Medicine, Medical Subject Headings
PFA: Partial foot amputation
PRISMA: Preferred Reporting Items for Systematic Reviews and Meta-Analyses
QoL: Quality of life
RR: Relative risk
SF-36: Medical outcomes short form 36
SIP: Sickness impact profile
TMA: Transmetatarsal amputation
TTA: Transtibial amputation
